# 

**Published:** 1919-04-19

**Authors:** 


### TUBERCULOSIS TOPICS.

### Winsley Sanatorium.

At the recent annual meeting of this institution it was stated that the number of patients received during the year amounted to 500. The total expenditure amounted to £9,728, which worked out at a weekly cost per bed of £1 19s. 10¾d. The expenditure on repairs and renewals had, however, been restricted. The total number of patients treated at this institution since its inception is now over 4,000. Winsley Sanatorium, which serves three counties, has indeed been in existence so many years that it is surprising to find that such things as a house for the senior resident medical officer, better housing for the nursing staff, and a cottage for the engineer, have not yet got beyond the stage of aspirations.

### Consumptive Soldiers.

A special committee has been set up by the Local Government Board and the Ministry of Pensions to consider and report upon the immediate practical steps which should be taken for the provision of residential treatment for discharged soldiers and sailors suffering from pulmonary tuberculosis, and for their reintroduction into employment, especially upon the land. Major Astor has been asked to act as chairman. The number of discharged tuberculous men awaiting residential treatment at the end of last February was approximately 470. In one large administrative sanitary area known to us a tuberculous soldier can be sure of being admitted to an institution, if he is willing, in about three weeks. But, as it was phrased, a civilian consumptive is "slipped in now and again." The plight of these latter, who pay their fourpences, but have had no taste of ninepences or hotels or anything else, is rapidly arousing indignation and discontent.

### New Journal for Tuberculosis.

Under the title *Tubercle* a new special journal is proposed to be issued next autumn from the publishing house of Messrs. Bale, Sons & Danielsson. The names of Drs. Egbert Morland and Claude Lellingston on the provisional editorial staff are a guarantee of painstaking and scholarly production. Somewhat of a feature will be made of abstracts from foreign special literature, while it is hoped to make the new publication a medium for publication of high-class original matter. However, some measure of lay appeal will not be excluded. We wish this new venture every success, and consider there is room for it and a great chance. It will be a monthly, the annual subscription 25s.; the address is 83 Great Titchfield Street, W. 1.

### A Mistake in the Plan.

The Norfolk Sanatorium Benefit Sub-Committee lately presented a report to their main body desiderating a farm colony for consumptives. They considered that it would provide for the requisite after-care of these patients. The proposal was for a farm to be taken, situated near a railway station and managed by a skilled superintendent of outdoor work and by a hospital-trained matron. The Insurance Committee's medical consulting officer would visit the institution, no resident medical officer being considered necessary. This last point is where an error of vital importance is made. Take the two best-known farm colonies for consumptives; these are near Edinburgh, and a lately established one in Cambridgeshire. At both these a resident medical man has supreme control of the institution. We fear that if the Norfolk Insurance Committee sticks to the idea of dispensing with resident medical control, the doubts of one of its members (as reported in the *Eastern Daily Press*) as to the amount of work that would be done, will soon be justified.

### PASSING EVENTS AND TOPICS.

### Newspapers as Germ Carriers?

In connection with the return of the influenza epidemic, a question was recently raised as to whether the present prevailing and largely increased custom of selling used newspapers to retailers for the purpose of wrapping up goods may not be in some way responsible for the spread of the disease. It is well known that books are "good" germ-carriers, and when it is remembered that the convalescent patient turns to the daily and weekly paper to while away the hours of his enforced idleness, the suggestion made may perhaps be worth consideration.

### The Institution's Rationing Register.

Those institutions, and we suspect they are few in number, which keep the register of consumption as directed by the Food Minister, will find their work lightened by recent Orders of the Ministry. Meat coupons need no longer be collected from non-residents, and restrictions as regards margarine have been removed. It is interesting to see the wild rush of competition in respect of margarine immediately the Food Controller lifts his hand; we earnestly hope that other articles as they cease to be controlled will be affected in the same welcome way.

---

A war memorial hospital, with forty-eight beds and the necessary staff accommodation, is to be erected at High Wycombe.

Nearly 13,000 "graduate nurses, nurses' aides, practical nurses, and volunteers" were assigned by the American Red Cross to fight the epidemic of influenza through which the country has just passed.

Mitcham, which had to be content with a hand-ambulance, is now in possession of a motor vehicle, the gift of the Showmen's Guild.

The Fur Trade Red Cross Appeal has resulted in a cheque for £23,453, which represents an effort extending over only four months among firms directly associated with the trade.

### Matrons' and Sisters' Appointments.

Hackney Union Infirmary, Homerton.—Miss Mary Irving and Miss Ruth Clara Dane have been appointed ward sisters. Miss Irving was trained at the Selly Oak Infirmary, Birmingham; has been staff nurse at the Lincoln City Hospital; sister at Keighley Infirmary; ward and theatre sister at Bradford Infirmary; and nursing sister at the Pensioners' Hospital, Birmingham. Miss Dane was trained at the Hackney Union Infirmary, and has done private and war nursing.

Maternity and Child Welfare Centre, Kilmarnock.—Miss M. B. Jack has been appointed matron. She was trained at the Kilmarnock Infirmary, at the Glasgow Royal Maternity Hospital, and at the Royal Asylum, Gartnavel, Glasgow. Miss Jack acted as theatre and surgical sister at the Kilmarnock Infirmary, and holds the certificates of the Central Midwives Board and of the Medico-Psychological Association.

Mauldeth Hospital for Incurables, Stockport.—Miss Kate Jenkins has been appointed matron. She was trained at the Derbyshire Royal Infirmary, where she was later sister, and she has been night sister, assistant matron, and temporary matron at the Stockport General Infirmary.

The Lady Home Cottage Hospital, Douglas, Lanarkshire.—Miss Margaret A. Hewetson has been appointed matron. She was trained at the Wolverhampton and Staffordshire General Hospital, where she was later temporary charge sister; has been sister at the 1st Southern Hospital, Birmingham; and has also done private nursing. Miss Hewetson holds the certificate of the Central Midwives Board.

London Homœopathic Hospital, Great Ormond Street, W.C.—Miss Janie Tiddy and Miss A. E. Pavey have been appointed gynæcological and night sister respectively. Miss Tiddy was trained at the above hospital, since taking temporary sister's duty. Miss Pavey was trained at the Prince of Wales's General Hospital, Tottenham, has been ward and theatre sister at Walsall General Infirmary, ward and casualty sister at the Hôspital Anglo Français de Trèport, theatre sister at the Hospital for Women, Soho Square, sister-in-charge of the Windlesham Court Auxiliary Hospital, pupil-housekeeper at the Hospital for Sick Children, Great Ormond Street, and temporary night superintendent at the Bethnal Green Infirmary.

West Kent General Hospital, Maidstone.—Miss Winifred M. Furze has been appointed lady superintendent. She was trained at University College Hospital, where she remained as sister. In 1914 she was appointed assistant matron, and the next year was given charge of the military extension block, which posts she has held up to the present time.

Tuberculosis Hospital, Fairfield House, near York.—Miss Edith Newsome has been appointed matron to this institution, which is shortly to be opened. She was trained at Portsmouth Infirmary and for midwifery at University College Hospital, holiday sister at the Dudley Union Infirmary, charge nurse at the London County Council Special Schools, E. Dulwich, health visitor and inspector of midwives for the County of Warwick, lecturer on nursing, hygiene and infant-care at the Gloucester School of Domestic Science, superintendent nurse at Worcester Union Infirmary, and matron of the New Hall Hey Auxiliary Military Hospital, Rawtenstall. Miss Newsome holds the certificate of the Central Midwives Board, certificate for hygiene from the Royal Institute of Public Health, is the author of "Home Nursing for V.A.D.s," is a member of the Council of the Royal British Nurses' Association, and has been mentioned in despatches.

Princess Alice Memorial Hospital, Eastbourne.—Miss C. Innes Griffin has been appointed matron. She was trained at Westminster Hospital, has been a member of Queen Alexandra's Imperial Military Nursing Service, and has been sister and matron at the Military Hospital, Eastbourne.

Lanark District Asylum, Hartwood.—Miss M. P. Jeffrey has been appointed matron. She was trained at Edinburgh District Asylum, Bangor, and at the Western Infirmary, Glasgow, and has since been assistant matron at Dykebar War Hospital, Paisley. Miss Jeffrey holds the certificate of the Medico-Psychological Association.

### The College of Nursing.

### (Limited by Guarantee.)

LIVERPOOL CENTRE.

(Hon. Sec.: Miss C. Worsley, Infirmary for Children.)

An interesting lecture was delivered on the 9th inst. to a large audience of members of the Liverpool Centre of the College of Nursing by Lieut.-Colonel Kelly on "The Eastern Front." The lecture was followed by a members' meeting to discuss the representation of the Centre on the College of Nursing Council. Miss Cummins, although with some reluctance, again yielded to the great pressure which was unanimously put upon her to stand again for nomination.

### Nominations for New Council.

Members of the College are reminded that if they wish to make a nomination for the new Council to be elected next month their nomination paper, a copy of which, with directions, should have already reached them, must be returned to the College, 7 Henrietta Street, W. 1, by Thursday, April 24.

### The Book Market.

LIST OF NEW BOOKS RECEIVED FROM THE FOLLOWING PUBLISHERS:—

Henry Frowde and Hodder and Stoughton: "Manual of Bacteriology." By Robt. Muir, M.A., M.D., and James Ritchie, M.A., M.D. 16s. net. "Orthopædic Effects of Gunshot Wounds and their After-Treatment." By G. W. Dow, M.B., B.S. 7s. 6d. net.

Wm. Smith and Son, Aberdeen: "Bydard Poems of War and Peace." By John Mitchell. 4s. 6d. net.

John Murray: "The Adventure of Life." By Robt. W. MacKenna, M.A. 6s. net. "Bodily Deformities." By the late G. J. Chance, F.R.C.S. Vol. II. Edited by John Poland, F.R.C.S. 18s. net.

John Wright and Sons, Ltd.: "Pye's Surgical Handicraft." Edited by M. H. Clayton-Greene, B.A., M.B., etc. 21s. net.

Cassell & Co., Ltd.: "The Doctor in War." By Woods Hutchinson, M.A., M.D. 7s. 6d. net. "Elements of Surgical Diagnosis." By Sir Alfred Pearce Gould, K.C.V.O., M.S. Lond., F.R.C.S. Eng.; and Eric Pearce Gould, M.A., M.Ch. Oxon., F.R.C.S. Eng. (Fifth Edition Revised.) 12s. 6d.

University of London Press: "Mental Disorders of War." By J. Lepine. Edited by C. A. Mercier, M.D. 7s. 6d. net. "Wounds of the Pleura and Lung." By Grégoire and Courcoux. Edited by C. H. Fagge, M.S. 7s. 6d. net. "Electro-Diagnosis of War." By Zimmern and Perol. Edited by E. F. Cumberbatch, M.A., 7s. 6d. "Diabetes of the Locomotor Apparatus." By Aug. Broca. Edited by Sir Robert Jones, F.R.C.S. 7s. 6d. net.

T. Werner Laurie, Ltd.: "A Textbook of Sex Education for Teachers and Patients." By W. M. Gallaghan. 6s. net.

